# Sensor Networks: Physical and Social Sensing in the IoT

**DOI:** 10.3390/s23031451

**Published:** 2023-01-28

**Authors:** Suparna De, Klaus Moessner

**Affiliations:** 1 Department of Computer Science, University of Surrey, Guildford GU2 7XH, UK; 2 Faculty of Electronics and Information Technology, Chemnitz University of Technology, Str. der Nationen 62, 09111 Chemnitz, Germany

Advances made in the Internet of Things (IoT) and other disruptive technological trends, including big data analytics and edge computing methods, have contributed enabling solutions to the numerous challenges affecting modern communities. With Gartner reporting 14.2 billion IoT devices in 2019 [1] and, according to some reports [2], a projected 30.9 billion devices to be deployed by 2025 in areas like environment monitoring [3], smart agriculture [4], smart healthcare [5] or smart cities [6], one could be tempted to think that most related issues are already resolved.

However, there remain practical challenges in large-scale and rapid deployment of sensors for diverse applications, such as problems affecting siting optimization methods and participant recruitment and incentive mechanisms. On a higher level, the deluge of data sources that drive the IoT phenomenon grows every day. With the rise of smartphone-enabled citizen sensing data via social networks or personal health devices, as well as with increasing connectedness in transport, logistics, utilities, or manufacturing domains, this range and complexity of available data calls for even more advanced data processing, mining and fusion methods than those already applied.

The goal of this Special Issue was to solicit high-quality original papers aimed at demonstrating effective and efficient deployment of sensor networks in the IoT, encompassing both physical and virtual sensor networks (through modelling) as well as social networks. Related issues of data processing, in addition to challenges of the fusion and visualisation of the resultant IoT big data, are also reflected in the published papers. This Special Issue consists of 11 papers. All submissions were strictly and thoroughly peer-reviewed by experts. These submissions cover many of the relevant research issues. In the following sections, we summarize these articles and highlight their major contributions.

In the article entitled “egoDetect: Visual Detection and Exploration of Anomaly in Social Communication Network”, Pu et al [7]. present a visualisation system for the analysis of anomalies in social graphs. Moreover, the authors design a novel glyph to explore an ego’s topology and the relationship between egos and alters. The proposed unsupervised method addresses the lack of labelled data in social networks, and the functionality of the developed system is demonstrated on a real-world operator’s call record dataset.

The article entitled “Recognizing Context-Aware Human Sociability Patterns Using Pervasive Monitoring for Supporting Mental Health Professionals”, contributed by Moura et al. [8], presents a proposal to detect context-aware sociability patterns. This would enable the identification of patterns in the periods of day in which users socialize, while also supporting the detection of abnormal behaviour and changes in daily routine. The solution presented does not detect, classify or predict mental health problems, but aims to identify situations of interest that can be further explored by mental health professionals. The work is evaluated using a well-known dataset of StudentLife, with interesting and logical results, as the identified sociability patterns show a strong positive correlation with individuals’ social routine. The detection of behavioural changes is important to mental health professionals and may indicate the occurrence of mental disorders.

Zurbuchen et al. [9], in their article “A Machine Learning Multi-Class Approach for Fall Detection Systems Based on Wearable Sensors with a Study on Sampling Rates Selection”, present a fall detection system (FDS) using an inertial measurement unit worn at the waist and evaluated with a public dataset. Their work extends the application of machine learning classification algorithms to a multi-class problem and an investigation into the effect of the sensors’ sampling rate on the performance of the FDS is performed, finding that the sampling rate of 50 Hz is generally sufficient for an accurate detection.

The article entitled “IoTCrawler: Challenges and Solutions for Searching the Internet of Things” by Iggena et al. [10] addresses the issues of the interoperability of IoT solutions and data fragmentation. The proposed IoT search framework, IoTCrawler, connects existing solutions and provides solutions for crawling, indexing and searching IoT data sources. A comprehensive evaluation combined with real-world case studies showcase the validation of the developed framework.

The paper entitled “Energy Management Expert Assistant, a New Concept” by Linan-Reyes et al. [11] presents a detailed report on a real-world deployed (2 years of testing, before full deployment) home energy management system that integrates the emerging technologies of IoT, AI, big data and expert systems for a home assistant, through a multi-objective optimization (MOP) problem. The resultant deployed system presents an interactive platform for optimized energy consumption in the home, which is both efficient and comfortable, while also improving security.

The topical theme of user device location privacy is explored in the paper “Perturbed-Location Mechanism for Increased User-Location Privacy in Proximity Detection and Digital Contact-Tracing Applications” by Lohan et al. [12], which presents perturbation-based location privacy protection, applied to location-based and proximity-based services (e.g., COVID-19 contact tracing). The approach is validated with simulation-based results in multi-floor building scenarios, enabling devices to adjust the accuracy level for location sharing with service providers.

The predictive maintenance of sensors is addressed in the article entitled “Providing Fault Detection from Sensor Data in Complex Machines That Build the Smart City” by Gascón et al. [13] through a case study of the application of a scheme of sensor data pre-processing and feature extraction to fault identification and classification in a bill-counting machine. Feature extraction is performed using the Kullback–Liebler divergence measure, enabling the visualization of the differences between normal and failure operating states, followed by fault classification using a neural network.

The application of IoT-enabled services to smart tourism scenarios is explored in the article “An Architecture for Service Integration to Fully Support Novel Personalized Smart Tourism Offerings” by Sabbioni et al. [14]. The article presents an innovative architecture for smart tourism services by integrating event and place information with transport ticketing. It successfully blends a technology-assisted experience with human-centric interaction and personalization, applying these to the Italian part of a historical pilgrim path, the “Francigena way”.

The use of short texts from social networks as a data source in the IoT is the focus of the article entitled “Investigating the Efficient Use of Word Embedding with Neural-Topic Models for Interpretable Topics from Short Texts”, submitted by Murakami and Chakraborty [15]. The authors study eight neural topic models, using simulation experiments with several benchmark data sets to assess the effectiveness of fine tuning and pretrained word embedding in generating interpretable topics. The paper concludes that the additional fine tuning step improves the performance of the neural topic models, a measure assessed through the topic coherence and topic diversity metrics, with GloVE [16] as the pre-trained word embedding.

“A Novel Privacy Preserving Scheme for Smart Grid-Based Home Area Networks”, submitted by Ali et al. [17], presents a privacy-preserving method for smart grid-based home area networks (HAN). Using homomorphic Paillier encryption, Chinese remainder theorem, and a one-way hash function, the proposed approach enables the detection of replay and false data injection attacks.

A review article, entitled “Applications of Wireless Sensor Networks and Internet of Things Frameworks in the Industry Revolution 4.0: A Systematic Literature Review” by Majid et al. [18], completes this SI. The systematic survey focuses on research solutions and new techniques to automate industry 4.0. Among the research questions explored in this paper, prominent are those related to network intruders and network security attacks on the IoT and wireless sensor network (WSN) layers, with the authors proposing a taxonomy for these issues. Challenges related to adaptation to 6G, the environment, supply chain management and limited resources are analysed.
